# Acknowledgement

**Published:** 1911-09

**Authors:** 


### Acknowledgement

OUR thanks are due to the *News* for the loan of the picture of the late Dr. Phelps.

---

The BUFFALO MEDICAL JOURNAL will publish regularly, full transactions of any professional organisation in western New York, at cost with allowance for additional subscriptions. Personal notices, brief reports of society proceedings, and the like, are always welcome and will be published without charge.

Subscribers are requested to remit promptly for the fiscal year beginning with August 1911. This request is due in part to the fact that the publication of the JOURNAL involves the payment of cash; in part to the simplicity and small amount of time required to register a cash subscription, as compared to the greater trouble of an open account; largely to the stringency of the postal laws with regard to bona fide subscriptions, on which depends the allowance of mailable sample copies. The greater the number of paid subscriptions, the more we can extend the circulation and, thus, improve the quality of the JOURNAL.

Non-subscribers receiving the JOURNAL, are requested to become subscribers. While we would prefer draft or money order, stamps or check ($2.00) will be accepted.

---

A LAW affecting physicians of New York State became of force on September 1. It requires reports to the State Commissioner of labor of all cases of industrial poisoning under treatment. The sections defining the duties of the physician and the penalty for non-observance are as follows:

Every medical practitioner attending on or called in to visit a patient whom he believes to be suffering from poisoning from lead, phosphorus, arsenic or mercury or their compounds, or from anthrax, or from compressed air illness, contracted as the result of the nature of the patient's employment, shall send to the commissioner of labor a notice stating the name and full postal address and place of employment of the patient and the disease from which, in the opinion of the medical practitioner, the patient is suffering, with such other and further information as may be required by the said commissioner.

If any medical practitioner, when required by this section to send a notice, fails forthwith to send the same, he shall be liable to a fine not exceeding ten dollars.

It shall be the duty of the commissioner of labor to enforce the provisions of this section, and he may call upon the state and local boards of health for assistance.

This act shall take effect September first, nineteen hundred and eleven.

---

Health Commissioner Fronczak, of Buffalo, has decreed that the public drinking cup, towel and combs and hair brushes must go. He advises the use of special paper towels, card board combs and paper cups for all public places. The old hanging hair brush is unequivocally condemned.

---

The Equitable Life Assurance Society of New York has established a conservation department.

Through that department and the medical department, the society will undertake to assist, by educational and perhaps other methods, in the conservation of human life.

The society will reach members and its medical representatives by means of an official publication dealing with this and other important subjects.

The society will extend such help as it legally may to the public authorities of the country in their efforts to improve the public health, and it will give its moral support (financial contributions cannot be made) to the general health conservation movement which has, in its various phases, reached nation-wide proportions, and accomplished most gratifying results in reducing life-waste.

